# Supplementary material for: The cristae modulator Optic atrophy 1 requires mitochondrial ATP synthase oligomers to safeguard mitochondrial function
Source: Nat Commun. 2018 Aug 24;9:3399. doi: 10.1038/s41467-018-05655-x (PMC6109181; doi:10.1038/s41467-018-05655-x)
Supplement: Supplementary file 1 — Supplementary Information [file 41467_2018_5655_MOESM1_ESM.pdf]

Supplementary Information

Quintana-Cabrera et al., “The cristae modulator Optic atrophy 1 requires mitochondrial ATP synthase oligomers to safeguard mitochondrial function”

Supplementary Figures

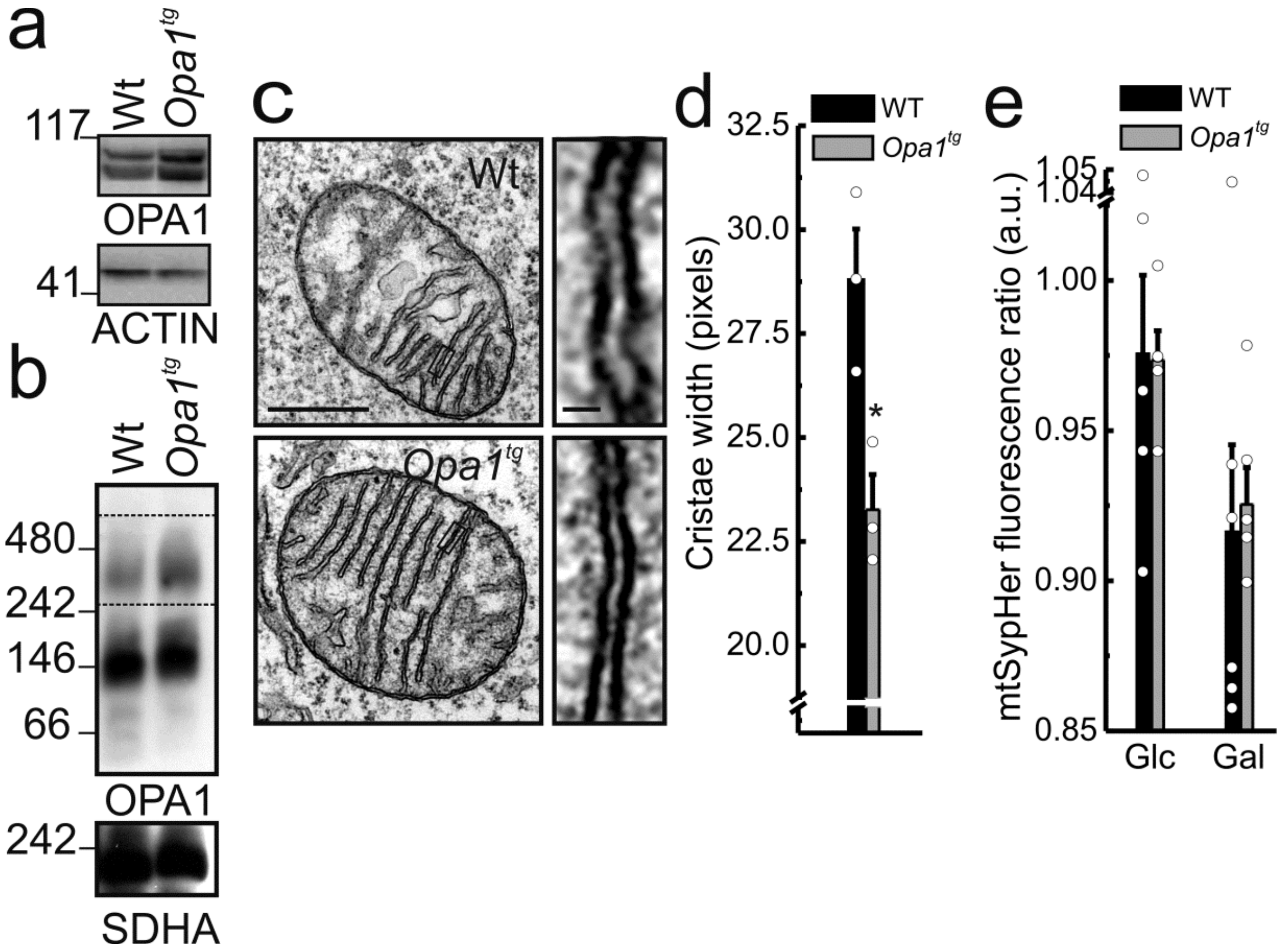

Supplementary Figure 1. OPA1 overexpression decreases cristae width without affecting basal matrix pH. Related to Fig. 1

(a) Protein extracts (30 µg) from cells of the indicated genotype were separated by SDS-PAGE and immunoblotted with the indicated antibodies.

(b) BNGE analysis of OPA1 HMW complexes. Protein extracts (30 µg) from cells of the indicated genotype were separated by BNGE and immunodecorated with the indicated antibodies. The dashed box indicate OPA1 HMW complexes.

(c) Representative EM micrographs of mitochondria in cells of the indicated genotype. Boxed areas are magnified in the right panels. Scale bars, 500 pixels in left and 20 pixels in right panels.

(d) Mean±SEM values of morphometric analysis of cristae width in 3 independent experiments as in (c). *$p<0.05$ in an unpaired two-sample Student's t test

(e) Matrix mySypHer fluorescence in cells of the indicated genotype cultured in media supplemented with the indicated hexose. Data represent mean±SEM of 5 independent experiments.

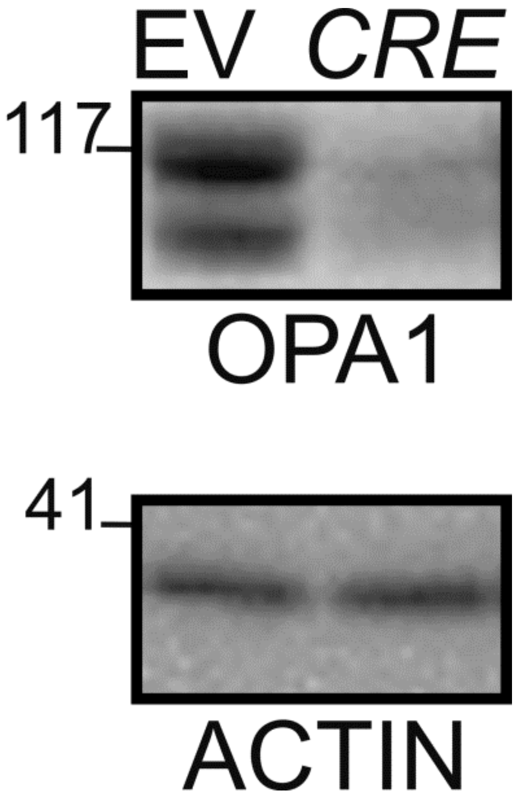

Supplementary Figure 2. Cre delivery deletes OPA1 in *Opa1*$^{flx/flx}$ MAFs. Related to Fig. 1

Protein extracts (30 µg) from *Opa1*$^{flx/flx}$ MAFs transfected with the indicated plasmids for 48 h were separated by SDS-PAGE and immunoblotted with the indicated antibodies.

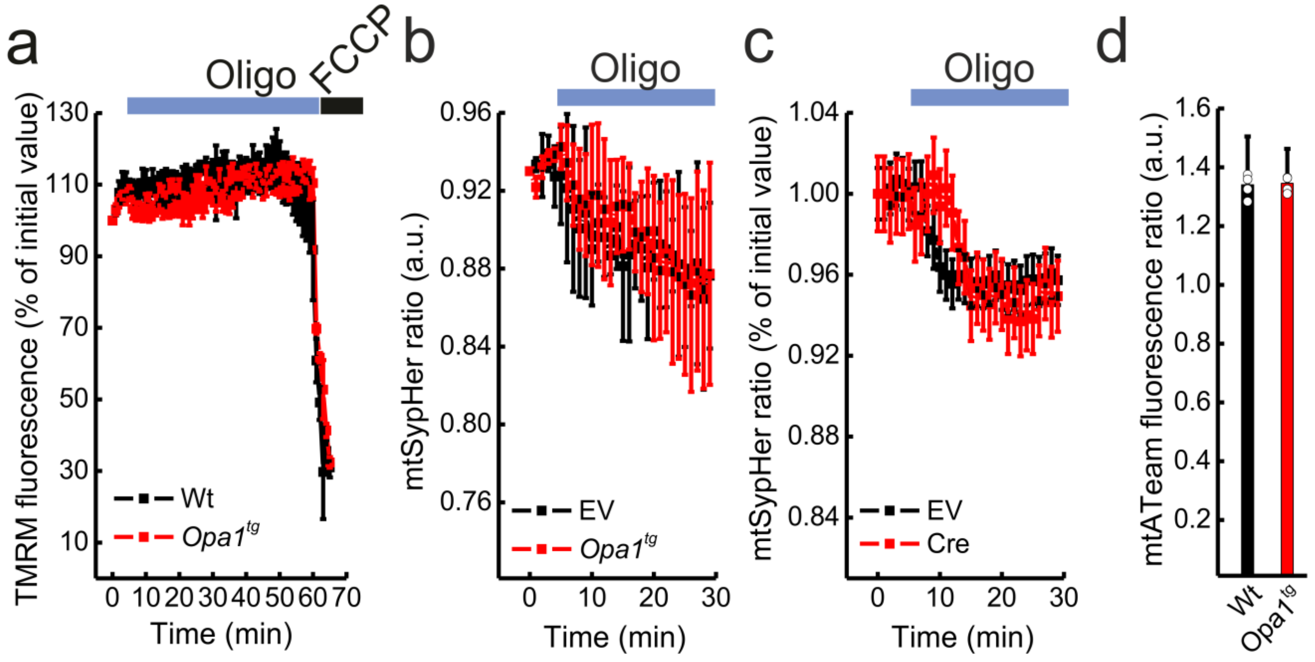

Supplementary Figure 3. OPA1 requires ATPase activity to sustain the mitochondrial electrochemical gradient, Related to Fig. 2

(a) Mean±SEM from at least 3 independent experiments of TMRM fluorescence real time imaging in cells of the indicated genotype. Where indicated, cells were treated with 1 µM oligomycin (Oligo) and 2 µM FCCP.

(b) Mean±SEM SypHer fluorescence ratios from cells of the indicated genotypes treated where indicated with 1 µM Oligo.

(c) Mean±SEM of at least 4 independent experiments of mtSypHer fluorescence ratios imaging in *Opa1*$^{flx/flx}$ cells, cotransfected with SypHer and the indicated plasmid (EV, Empty vector; Cre: Cre recombinase). Where indicated cells were treated with 1 µM Oligo.

(d) Basal mean±SEM mtATeam ratios from at least 4 independent experiments performed in cells of the indicated genotype incubated for 24 h in galactose medium.

.

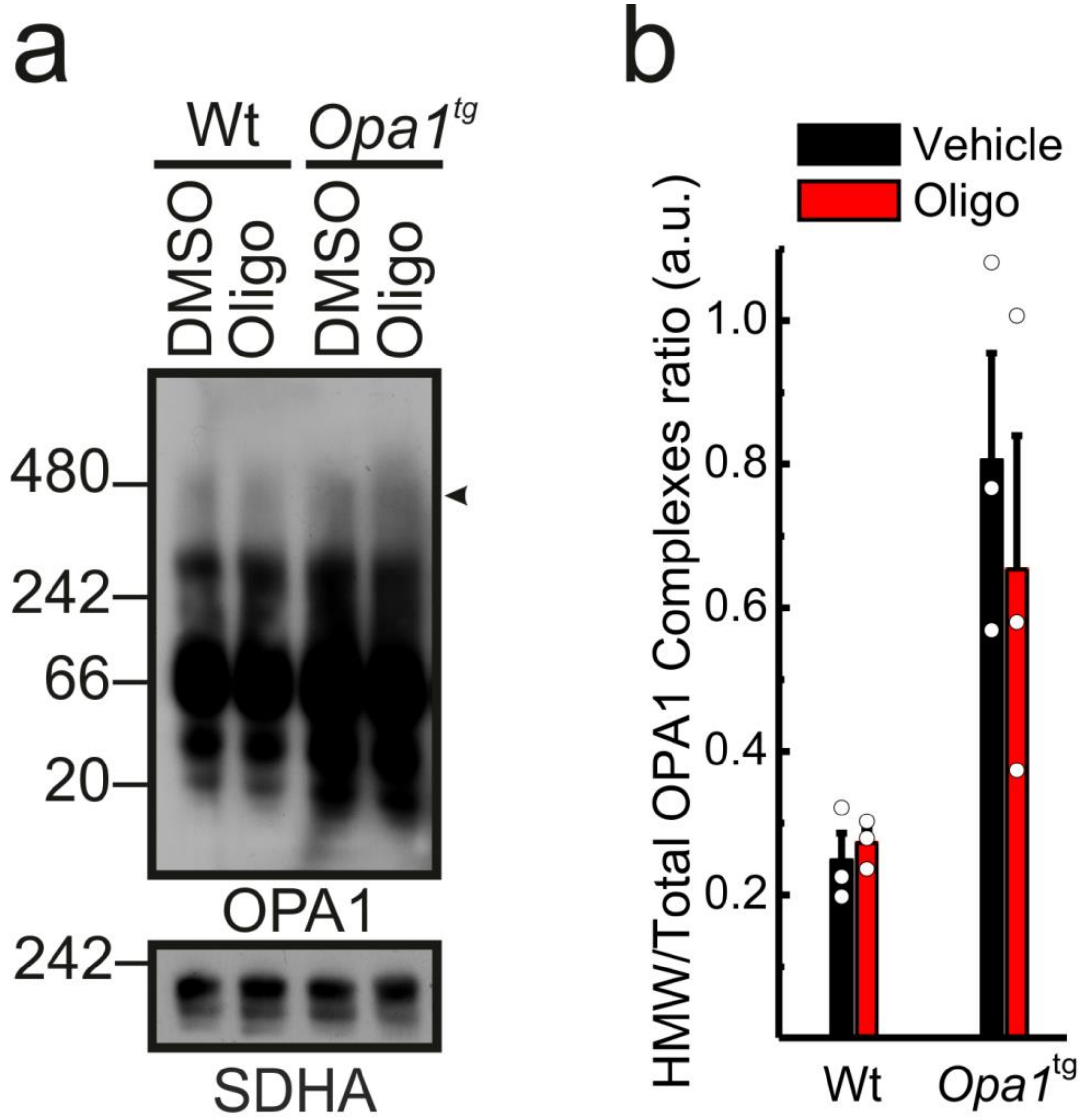

Supplementary Figure 4. Oligomycin does not destabilize OPA1 HMW complexes. Related to Fig. 2

(a) BNGE analysis of OPA1 HMW complexes. Protein extracts (40 µg) from cells of the indicated genotype treated where indicated with 1 µM oligomycin for 30 min were separated by BNGE and immunodecorated with the indicated antibodies. Arrowhead indicates OPA1 HMW complex. SDHA: succinate dehydrogenase A.

(b) Mean± SEM of densitometric analysis of OPA1 high molecular weight (HMW, arrowheads) complexes versus total OPA1 conformations in 3 independent experiments as in (a).

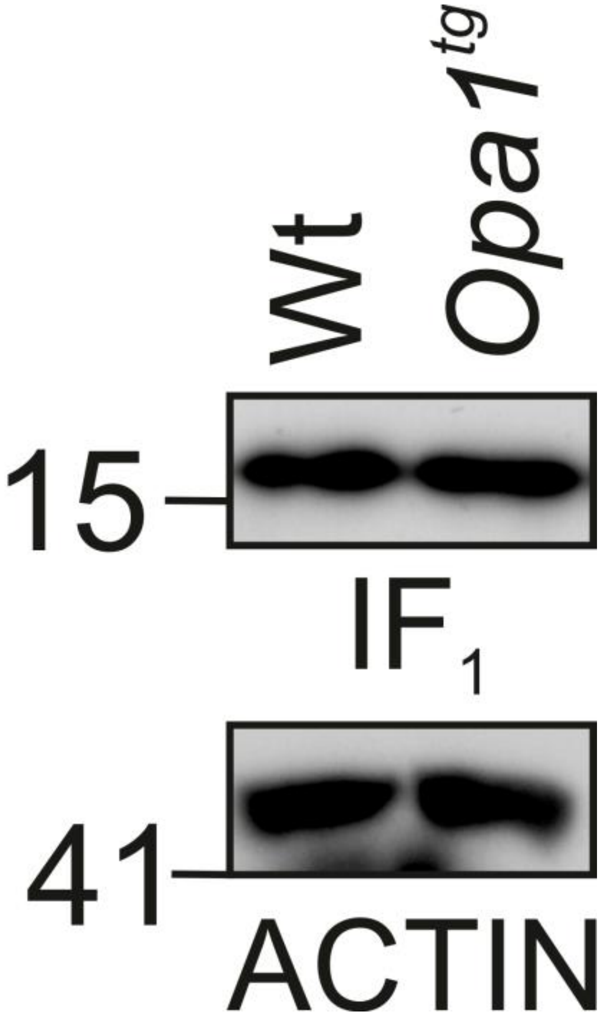

Supplementary Figure 5. IF_1 levels are comparable in WT and *Opa1^{tg}* MAFs, Related to Fig. 3

Protein extracts (30 μg) from WT and *Opa1^{tg}* MAFs were separated by SDS-PAGE and immunoblotted with the indicated antibodies.

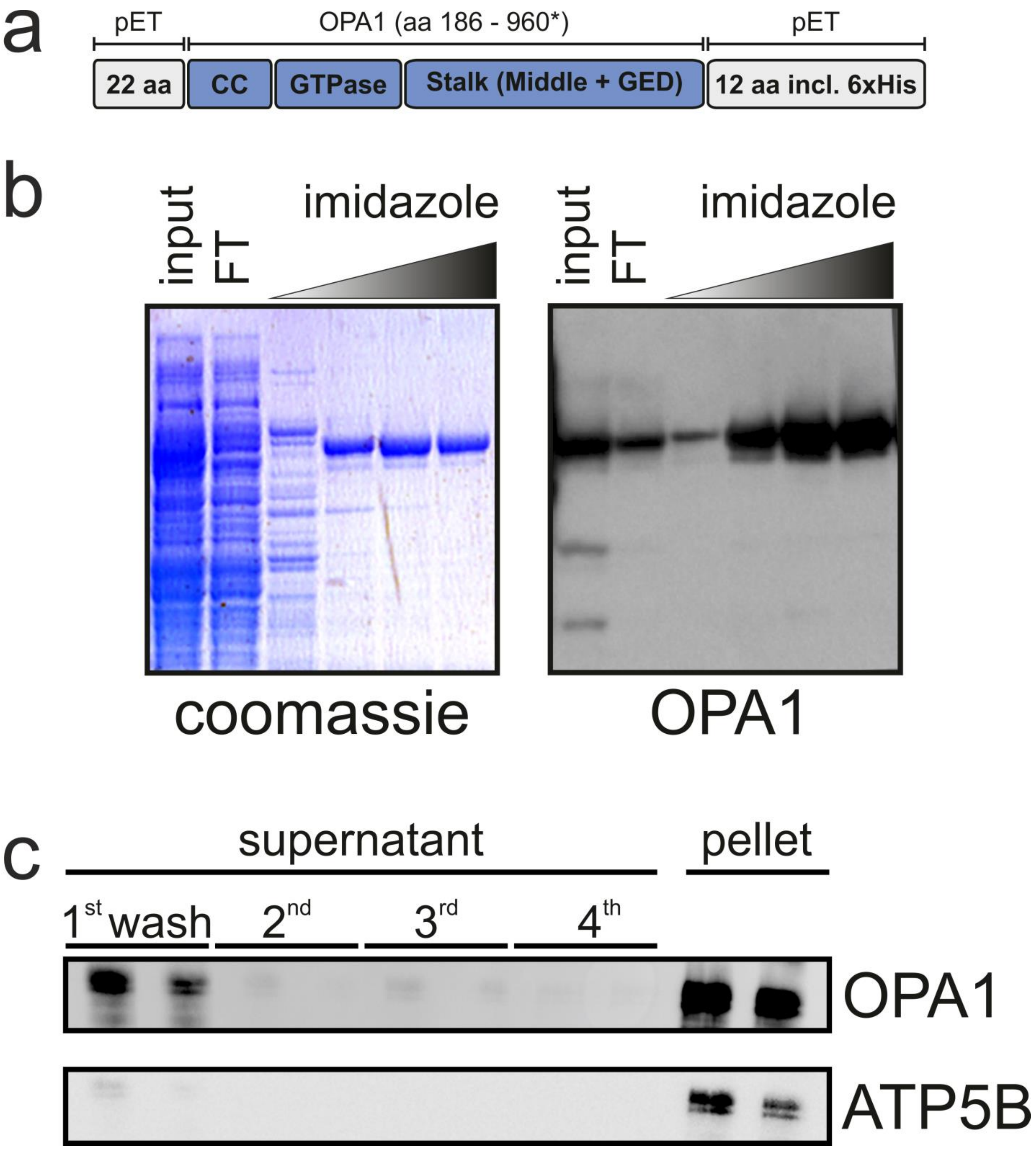

Supplementary Figure 6. Production, purification and incorporation of rOPA1 in proteoliposomes. Related to Fig. 5
(a) Cartoon of the produced recombinant OPA1 (rOPA1).
(b) Representative purification of rOPA1 analyzed by sampling at the indicated steps of the purification process and subsequent separation by SDS-PAGE. Proteins were visualized by immunoblotting with an anti-OPA1 antibody or Coomassie staining.
(c) Incorporation of rOPA1 in ATP synthase containing liposomes. Proteoliposomes incorporating purified ATP synthase (20 µg) were prepared in the presence of 20 µg rOPA1. Proteoliposomes were washed by centrifugation at 100,000xg. Proteins from the final pellet and the prior supernatants were separated by SDS-PAGE and immunoblotted using the indicated antibodies.

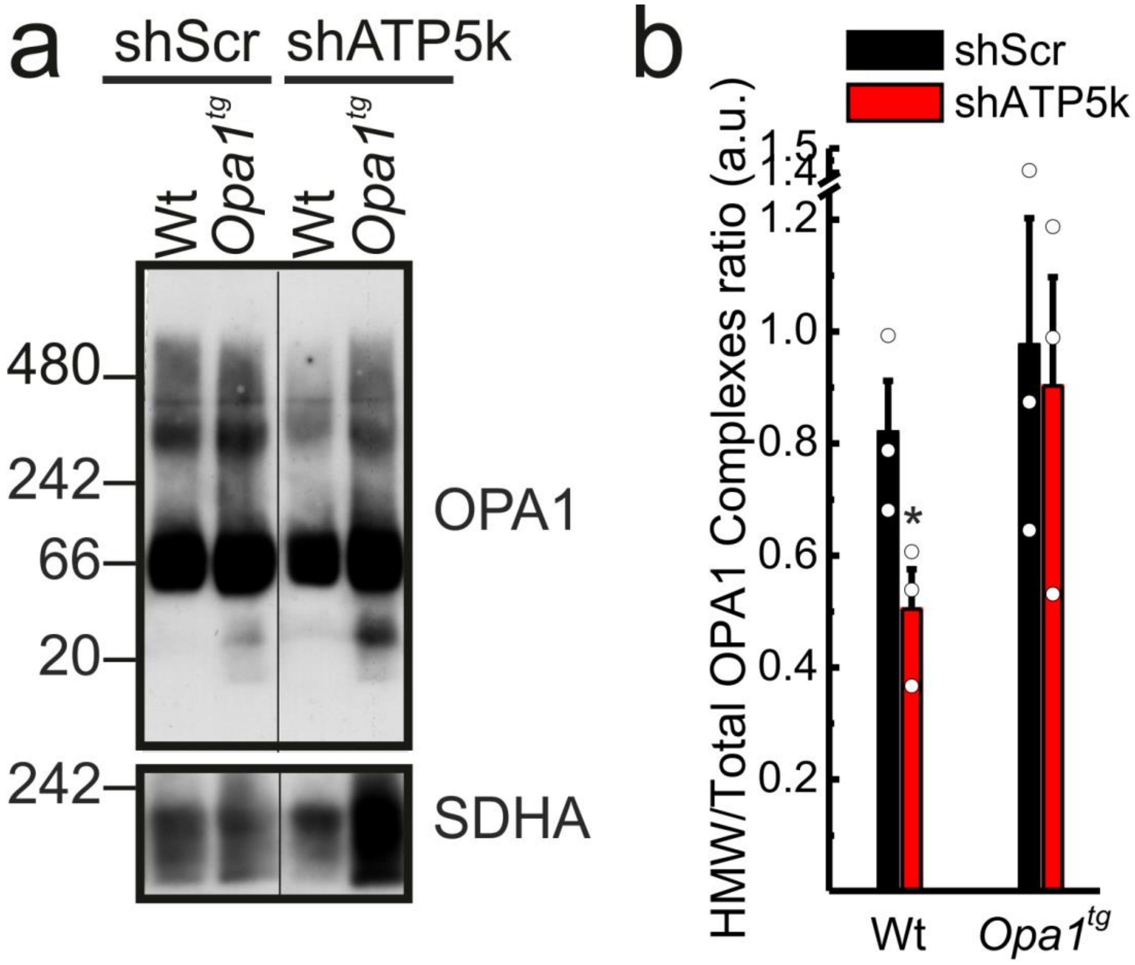

Supplementary Figure 7. ATP5k silencing destabilizes OPA1 oligomers in Wt MAFs. Related to Fig. 6
(a) BNGE analysis of OPA1 HMW complexes in protein extracts (30 µg) from cells of the indicated genotype transfected for 48 h with the indicated shRNA. SDHA: succinate dehydrogenase A.
(b) Mean±SEM of densitometric analysis of OPA1 HMW/CII ratio, calculated from 3 independent experiments as in (a).

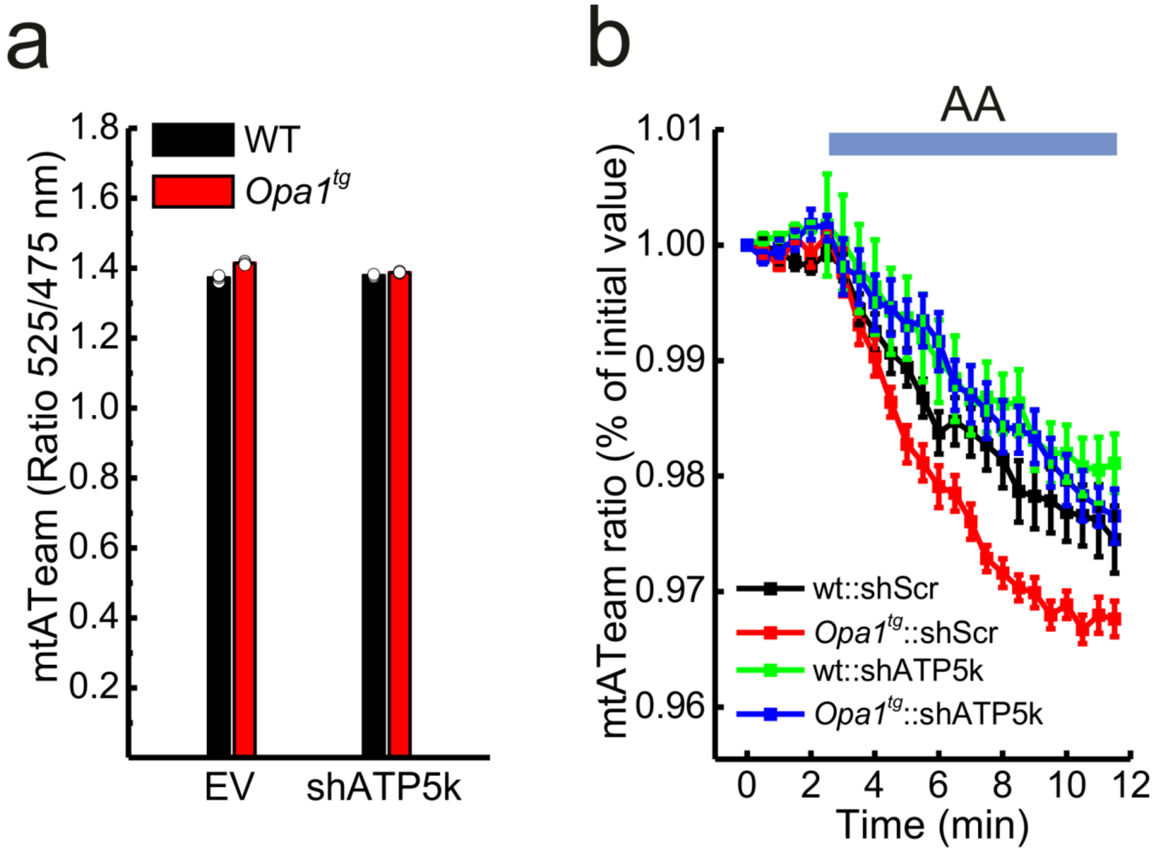

Supplemental Figure 8. OPA1 requires ATP5k to promote ATPase activity, Related to Fig. 7.

(a) Basal mtATeam mean±SEM FRET fluorescence emission ratios (525/475 nm) from at least 3 independent experiments performed in MAFs of the indicated genotype transfected with the indicated shRNA for 72 h.

(b) Mean±SEM normalized real time mtATeam FRET fluorescence analysis from 3 independent experiments as in (a). When indicated, cells where treated with 2 μM AA.

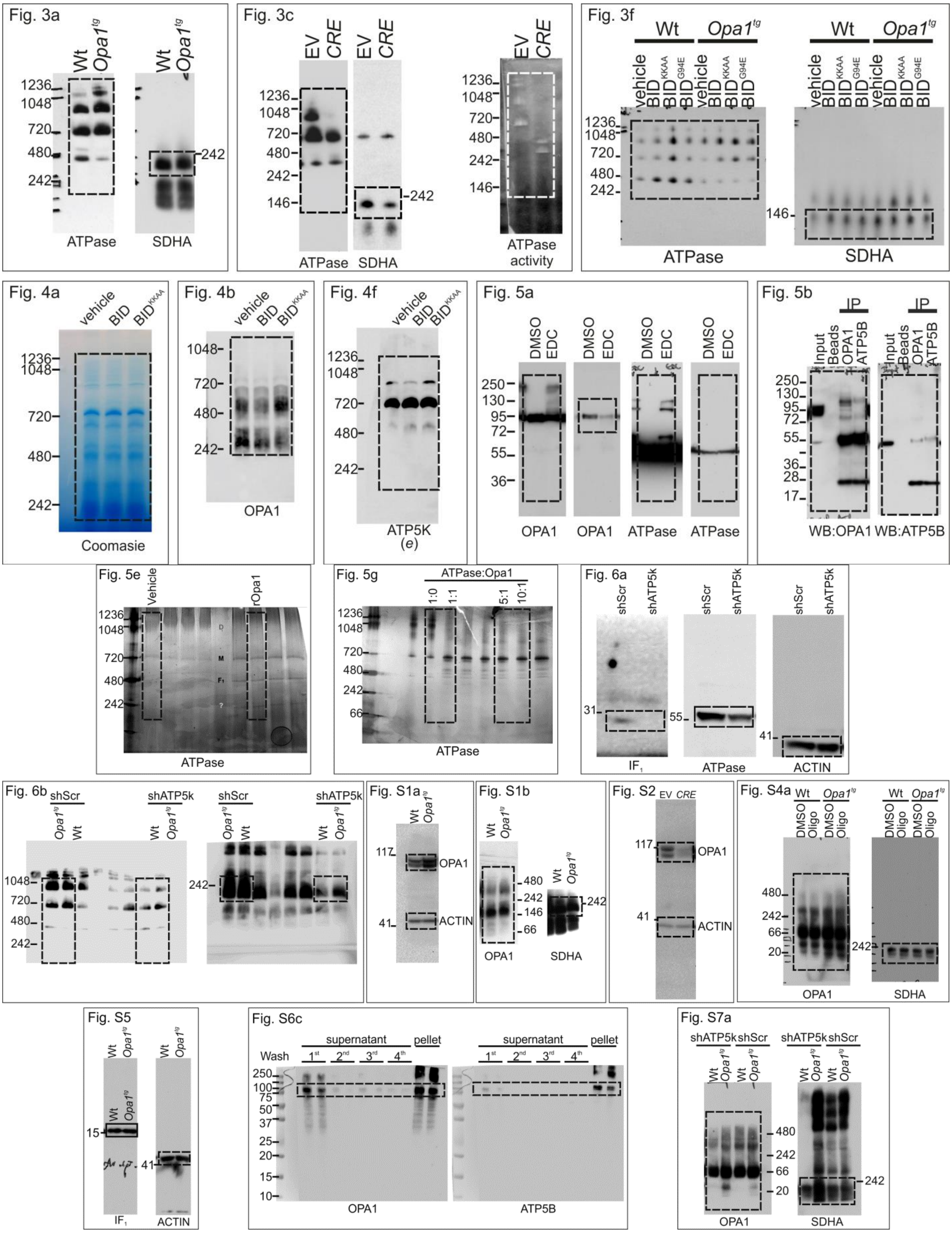

Supplemental Figure 9. Uncropped blots

Uncropped blots of the areas indicated in the shown figures. If not added here, full blots are shown in the figures. Dashed boxes indicate the cropped area
