# Supplementary material for: The cristae modulator Optic atrophy 1 requires mitochondrial ATP synthase oligomers to safeguard mitochondrial function
Source: Nat Commun. 2018 Aug 24;9:3399. doi: 10.1038/s41467-018-05655-x (PMC6109181; doi:10.1038/s41467-018-05655-x)
Supplement: Supplementary file 3 — Description of Additional Supplementary Files [file 41467_2018_5655_MOESM3_ESM.pdf]

Description of Additional Supplementary Files

File Name: **Supplementary Movie 1**
Description: **Real time imaging of mitochondrial matrix ATP levels following complex III inhibition.** Cells of the indicated genotype expressing mtATeam1.3 were treated when indicated with 10µM antimycin A. The frame when antimycin A is added is indicated and held still for 10 frames. mtATeam1.3 FRET ratio is pseudocolored using a rainbow scale.

File Name: **Supplementary Movie 2**
Description: **Real time imaging of mitochondrial membrane potential levels following complex III inhibition.** Cells of the indicated genotype were loaded with 20nM TMRM and treated when indicated with 10µM antimycin A and 2 µM FCCP. The frames when antimycin A and FCCP are added are indicated and held still for 10 frames. Where indicated (KD sub e), ATP5k was silenced as described in the main text

File Name: **Supplementary Data 1**
Description: **List of ATP synthase subunits analyzed by MS in Figure 4.** Accession numbers are provided along with the description of the genes and proteins analyzed by MS.
