# Supplementary figures and images for: The cristae modulator Optic atrophy 1 requires mitochondrial ATP synthase oligomers to safeguard mitochondrial function
Source: Nat Commun. 2018 Aug 24;9:3399. doi: 10.1038/s41467-018-05655-x (PMC6109181; doi:10.1038/s41467-018-05655-x)

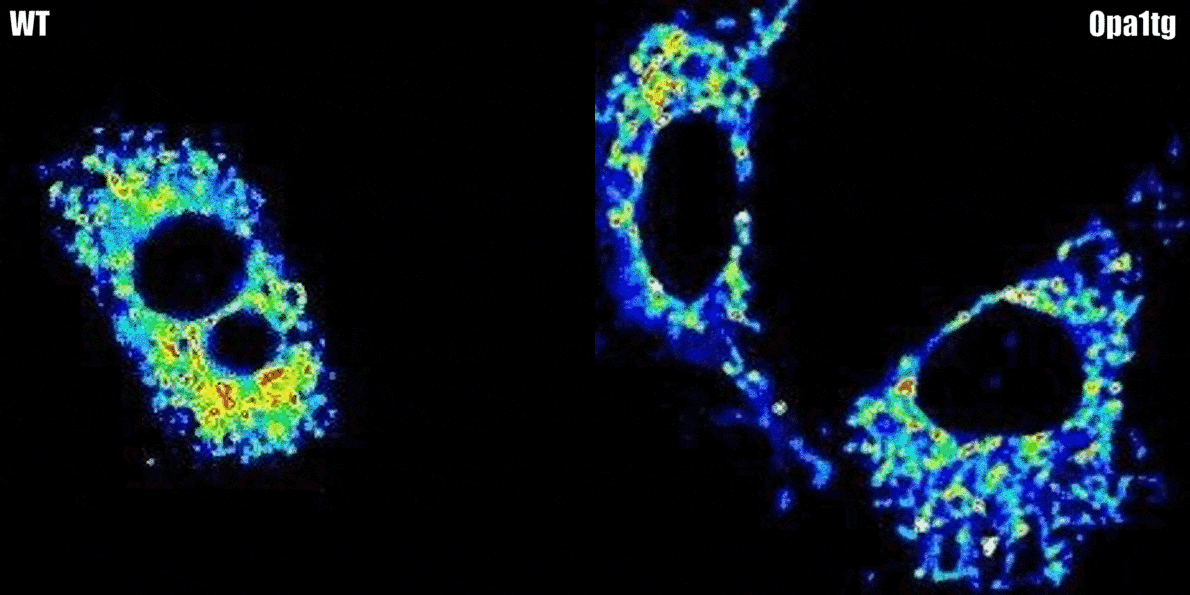

Supplement: Supplementary file 4 — Supplementary Movie 1 [file 41467_2018_5655_MOESM4_ESM.gif]

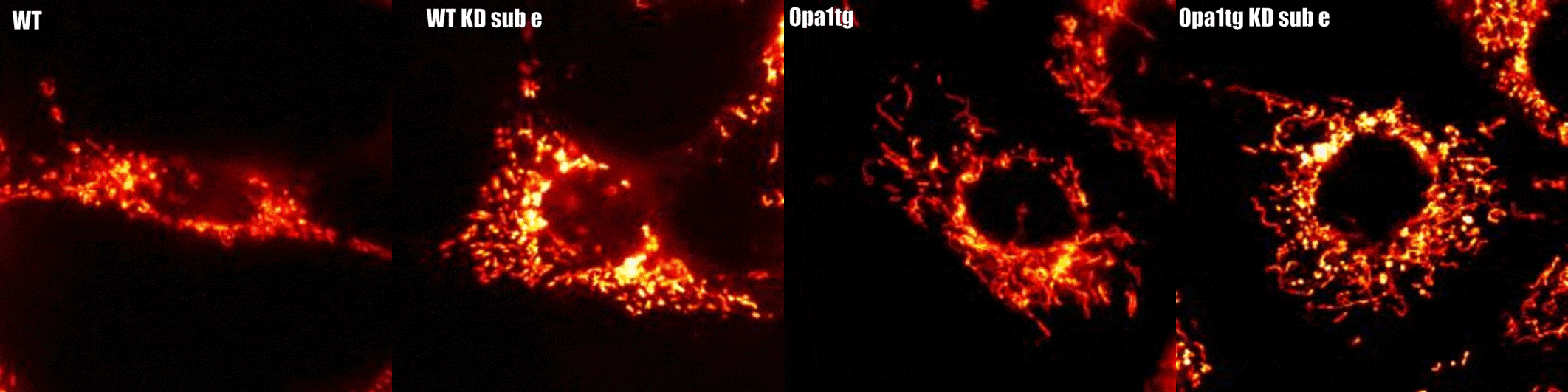

Supplement: Supplementary file 5 — Supplementary Movie 2 [file 41467_2018_5655_MOESM5_ESM.gif]
